# A computer vision approach to improving cattle digestive health by the monitoring of faecal samples

**DOI:** 10.1038/s41598-020-74511-0

**Published:** 2020-10-16

**Authors:** Gary A. Atkinson, Lyndon N. Smith, Melvyn L. Smith, Christopher K. Reynolds, David J. Humphries, Jon M. Moorby, David K. Leemans, Alison H. Kingston-Smith

**Affiliations:** 1 Centre for Machine Vision, Bristol Robotics Laboratory, University of the West of England, Bristol, BS16 1QY UK; 2 Centre for Dairy Research, School of Agriculture, Policy and Development, Earley Gate, University of Reading, Reading, RG6 6AR UK; 3 Institute of Biological, Environmental and Rural Sciences, Aberystwyth University, Gogerddan, Aberystwyth, SY23 3EE UK

**Keywords:** Animal behaviour, Animal physiology

## Abstract

The digestive health of cows is one of the primary factors that determine their well-being and productivity. Under- and over-feeding are both commonplace in the beef and dairy industry; leading to welfare issues, negative environmental impacts, and economic losses. Unfortunately, digestive health is difficult for farmers to routinely monitor in large farms due to many factors including the need to transport faecal samples to a laboratory for compositional analysis. This paper describes a novel means for monitoring digestive health via a low-cost and easy to use imaging device based on computer vision. The method involves the rapid capture of multiple visible and near-infrared images of faecal samples. A novel three-dimensional analysis algorithm is then applied to objectively score the condition of the sample based on its geometrical features. While there is no universal ground truth for comparison of results, the order of scores matched a qualitative human prediction very closely. The algorithm is also able to detect the presence of undigested fibres and corn kernels using a deep learning approach. Detection rates for corn and fibre in image regions were of the order 90%. These results indicate the potential to develop this system for on-farm, real time monitoring of the digestive health of individual animals, allowing early intervention to effectively adjust feeding strategy.

## Introduction

Accurate feeding of animals in the beef and dairy industries is important both for efficient production and to reduce the impact of cattle farming on the wider environment. Sustainability in the ruminant livestock sector involves efficient use of resources to deliver quality products (meat and milk) with minimal impact on the environment. By improving estimates of feed composition, digestibility, and digestive health, it will be possible to obtain:

- Efficient and more precise nutrient delivery to the animal, thereby reducing instances of overfeeding nutrients such as protein and improving productivity which will maintain competitiveness of home-produced livestock products.
- Minimised release of pollutants such as ammonia and urea (arising from poor utilisation of protein in the rumen and the animal) to land and water.

These important societal and economic targets are directly related to the mitigation of climatic impact of livestock and can be facilitated via the development of tools to support sustainable livestock production systems^1^.

Both under- and over-feeding of nutrients are inefficient and can lead to environmental, economic and welfare issues. Farm businesses cannot afford to waste expensive resources by feeding nutrients in amounts surplus to requirements^2^. Equally, it is relatively common for farm rations to perform under expectations: too much or too little of some components, poor mixing or poor sorting can lead to poor productivity, in addition to health and welfare problems^3^. Optimisation of feed for production ruminants is imperfect with discrepancies often arising, especially for precision feeding strategies that aim to minimise surplus nutrient supply. For fresh forage feeds, the general (“book”) dietary parameters may not agree with the actual values resulting from genenomic and environmental factors (often referred to as the “GxE effect”) and management^4^. Further, for total mixed rations (TMR) the nutritional values may be affected by on farm storage conditions^2^.

There is a current need for improved methods of assessment of feed use efficiency; especially ones that can be directly applied on a farm. Key issues are the presence of excess starch or too little effective fibre in the feed, which can lead to subclinical rumen acidosis^5^. However, the farmer's ability to make feed strategy decisions quickly is restricted by the time needed for off-site lab-based chemical analysis of feed and faeces and the lack of an appropriate method for determining the optimal level of effective fibre required. Equally, currently available on-farm diagnostics^6^, often relying on visual assessment, are not well positioned to make specific recommendations for remedial actions to halt losses in milk or meat production, or poor feed use efficiency. Rapid and accurate diagnosis of poor feed use efficiency will enable more effective dietary adjustments to be made to improve nutrient use efficiency and sustain production.

Given the above discussion, new proxies for feed adequacy are sought based on real-life practice that can be applied in a tool for better on-farm resource management. This paper proposes a method to parametrise faecal consistency as an indicator of gut health and diet fibre content using computer vision. This is a less subjective version of current on-farm visual assessment scores of faecal consistency^6–8^. A portable imaging system has been developed to include near-infrared (NIR) or visible light sources able to capture and analyse the three-dimensional appearance of samples and formulate an objective health score based on consistency. Further, machine learning has been applied to a database of training images to extract data related to the presence of undigested fibres and corn kernels in the samples adding to the health score above.

In addition to the immediate estimation of cattle digestive health, it is envisaged that, ultimately, this method will allow for the estimation of feed quality (e.g. protein and fibre concentrations and particle size). In combination with other technology such as NIR spectroscopy, the development of “on-farm visual analysis” as an additional automated diagnostic tool will significantly enhance the ability to make real-time feeding decisions. This will enable more precise, strategic feeding of individual animals (in a so-called “precision” farming manner) and herds for on-farm nutrient management to improve welfare, production and the environment.

It is hoped that, with the proposed system, it will be easy for farmers to frequently assess the digestive health of cattle on the farm with minimal training and cost outlay. The next section of this paper, Materials and methods, describes the bespoke hardware used for this project, and computer vision algorithms for faecal consistency scoring and grain/fibre detection. After this, a detailed Results and discussion section is presented before a Conclusion with consideration of limitations and potential future work.

## Materials and methods

This section first describes the hardware and methods used to acquire the necessary data for the two parts of the image analysis, i.e. consistency score estimation and corn kernel/fibre detection. It then furnishes the algorithms used to subsequently process the data.

### Data capture

The requirements for the data capture hardware were that:

- the system is relatively low cost;
- the system is easy to use and portable;
- the system is minimally affected by typical farm conditions including common changes to ambient light and temperature;
- fibres and corn are visible, where possible;
- the surface structure (e.g. three-dimensional roughness) associated with the scoring method can be extracted; and
- there is a manageable computational expense.

Note that corn kernels were used as a common test case—other undigested cereal grains will be considered in future work using a similar paradigm.

The method of photometric stereo (PS)^9^ was chosen as the basic hardware for data capture. This technique involves the capture of at least three 2D images of an object with different illumination directions, which are then used to determine the orientation of the surface of the sample (in the form of surface normal vectors) at each pixel on the target object/sample. The map of surface normals can be integrated into a depth estimate^10^. However, this step should only be used if necessary as it introduces distortions to the data due to the accumulation of noise and systematic artefacts in the reconstruction process^11^. Therefore, the adopted algorithm, as described below, uses the surface normal data as a means of encoding 3D information. The method was chosen due to its ability to obtain both 3D and 2D surface geometry simultaneously, its relatively low cost of off-the-shelf components and its high resolution; thus largely meeting the above requirements.

**Figure 1:**
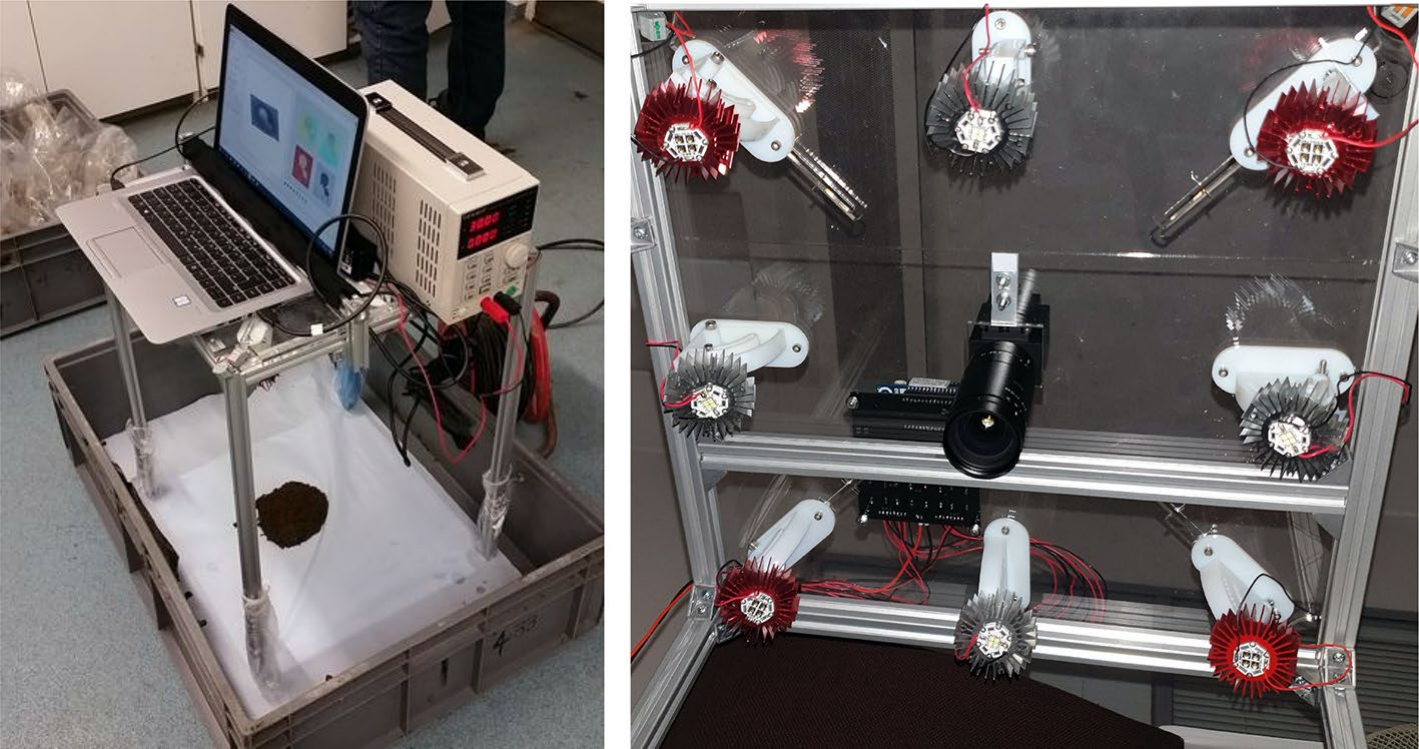
Photograph of the bespoke data capture rig. Left: during an actual sample data capture in laboratory conditions. Right: from below without power supply and laptop. Note that the camera and LEDs are not visible in the left image as they are underneath the computer/power supply.

**Figure 2:**
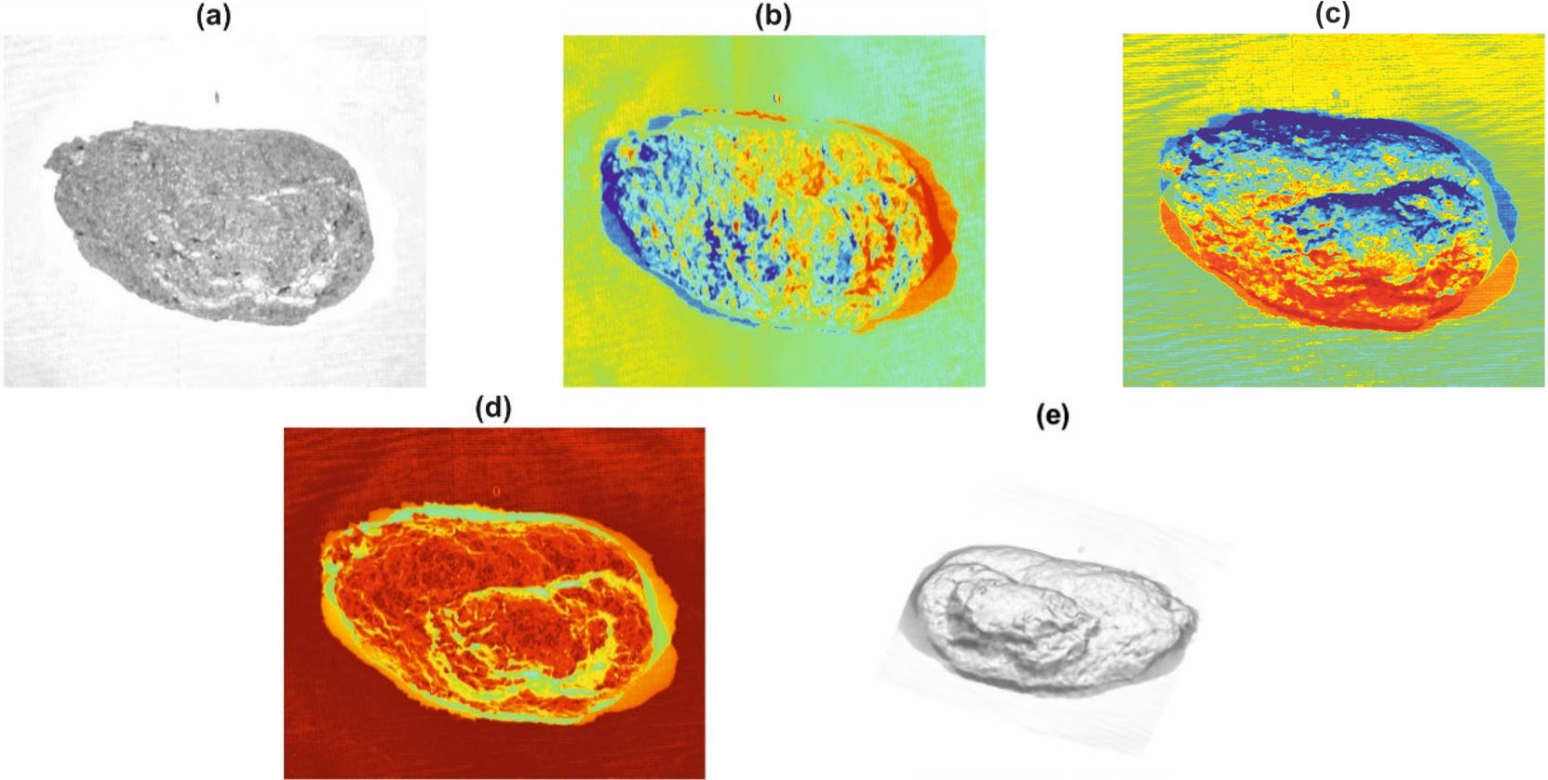
Sample data captured from the PS rig shown in Fig. 1. (**a**) Albedo. (**b**–**d**) $x$, $y$ and $z$ components of surface normals respectively. (**e**) Depth. NIR illumination was used for this figure.

For this project, a bespoke rig was constructed based on earlier work carried out by the authors for other applications^11,12^. The rig, shown in Fig. 1, consists of a USB3.0 Point Grey Grasshopper camera (both colour and monochrome NIR were used), four white LEDs and four NIR LEDs (850 nm). The lights are interfaced to a laptop computer (that can be bolted on top of the device) via an Arduino micro-controller and Python code. The code triggers the camera to capture images in turn with one image per illuminated light source. The Python code also processes the raw images to obtain albedo (reflectance) and surface normal data. An example of the recovered albedo, surface normals and depth is shown in Fig. 2. The algorithm either uses NIR or visible lights but not both simultaneously. Experiments determined that the NIR data appeared to give better results for 3D analysis but that the visible light offered superiority for some of the 2D aspects covered below.

### Scoring

This section describes an approach to score the health of a cow using the consistency of its faeces as an indicator of digestive function and health. In the literature, several researchers have attempted to standardise the parametrisation of manure structure using a score from 1 (very runny) to 5 (firm). While there is a large degree of subjectivity associated with this approach, it forms a well-established foundation on which to build a metric for this project^6,13^.

#### Concept

Based on consideration of the 2D and 3D appearance variation in samples of particular scores and the mechanics by which a sample drops to the ground, it was decided to parametrise each sample for this project using three metrics and then combine them into the overall score. The metrics are termed:

- Border metric
- Shininess metric
- Normal metric

The *border metric* parametrises the roughness or irregularity of the border of the sample as it appears on the ground, noting that a runny low-score sample would splatter widely, forming a rough border. The *shininess metric* describes the reflectance of the sample: lower scoring samples are more shiny (while it may seem more logical to assign higher scores to shinier samples, the equations/algorithm below are kept more compact when matte samples score more highly). Finally, the *normal metric* characterises the roughness of the 3D surface topography within the body of the sample itself. Referring to the literature^13^ it can be seen that the low-score samples are smoother than high score samples.

In summary, a sample with a high total score should correspond to high values of the normal metric but low values for border and shininess metrics. If we refer to the metrics for border, shininess and normal as $S_{B}$, $S_{S}$ and $S_{N}$ respectively, then we can pose the *total score*, $S$, as:

1 $$ S = \frac{{S_{N} }}{{S_{B} S_{S} }} $$

All data for this section were captured at the University of Reading Centre for Dairy Research (CEDAR) in October 2018. The full dataset is publicly available^14^. There was a total of twenty samples which had been frozen and then thawed for the data capture session. In each case, the thawed sample was stirred to restore original consistency as much as possible (although some liquid had escaped from the rest of the sample during the thaw) and 0.2 kg extracted and placed in a small pot. The sample was then dropped from a fixed height of approximately 1 m (to approximate the act of animal defecation) onto a white acrylic sheet covered in blue tissue paper. The capture rig was carefully placed above the sample and then used for data capture, as shown in Fig. 1.

To enable consistent conditions for experimentation, the data was captured indoors with the lights switched off and only minimal daylight reaching the sample from outside. However, a significant amount of ambient light was still present and seemed not to adversely affect results when compared to images taken in complete darkness (with a shroud over the capture hardware). To optimise the image quality, a different aperture was used for visible and NIR lights ($f1.8$ and $f4$ respectively). Experimental results later showed that NIR illumination gave superior results for the normals metric while visible illumination fared better for the border and shininess metrics when compared to human subjective evaluation.

**Figure 3:**
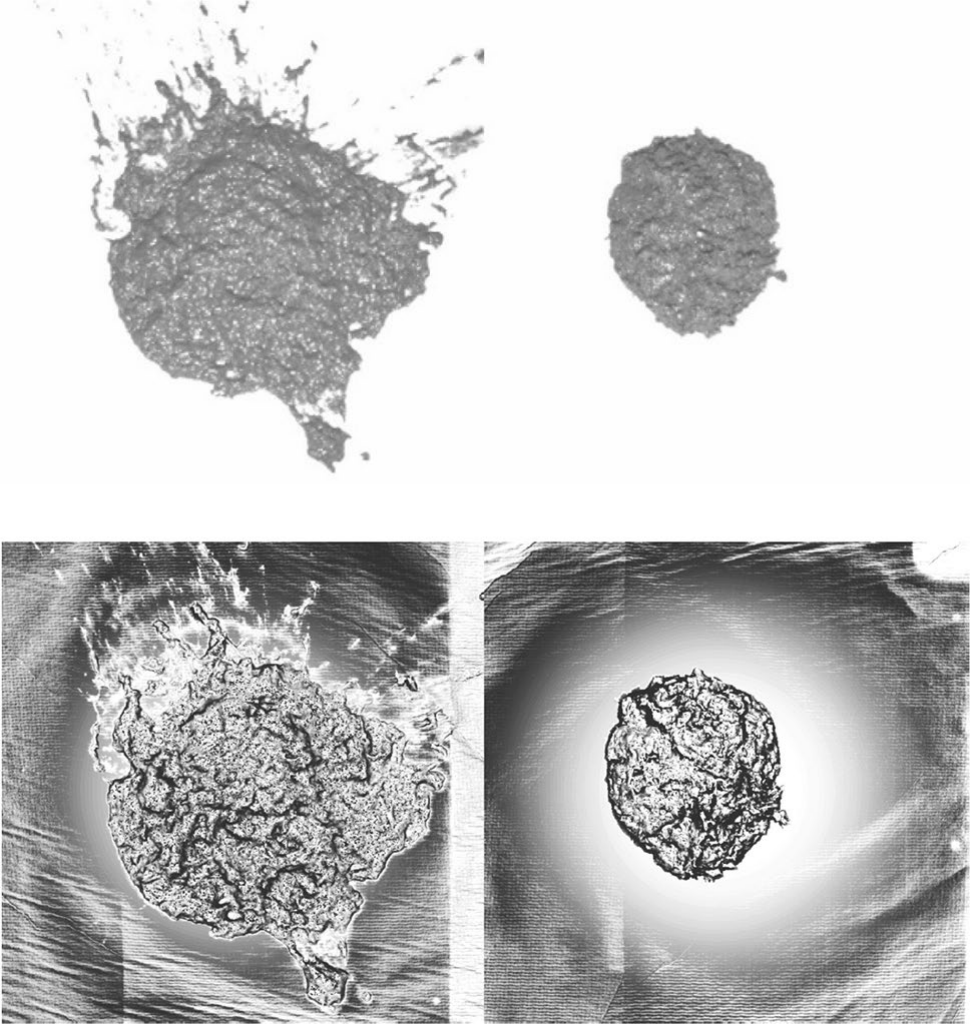
2D images (top) and 3D surface normals (bottom) of the lowest-scoring (left) and highest-scoring (right) samples.

For reference, Fig. 3 shows the visible light image and a rendering of the surface normals for the samples that later gave the lowest and highest scores respectively. These will be used in the next section as test cases to describe the algorithm.

#### Algorithm

**Figure 4:**
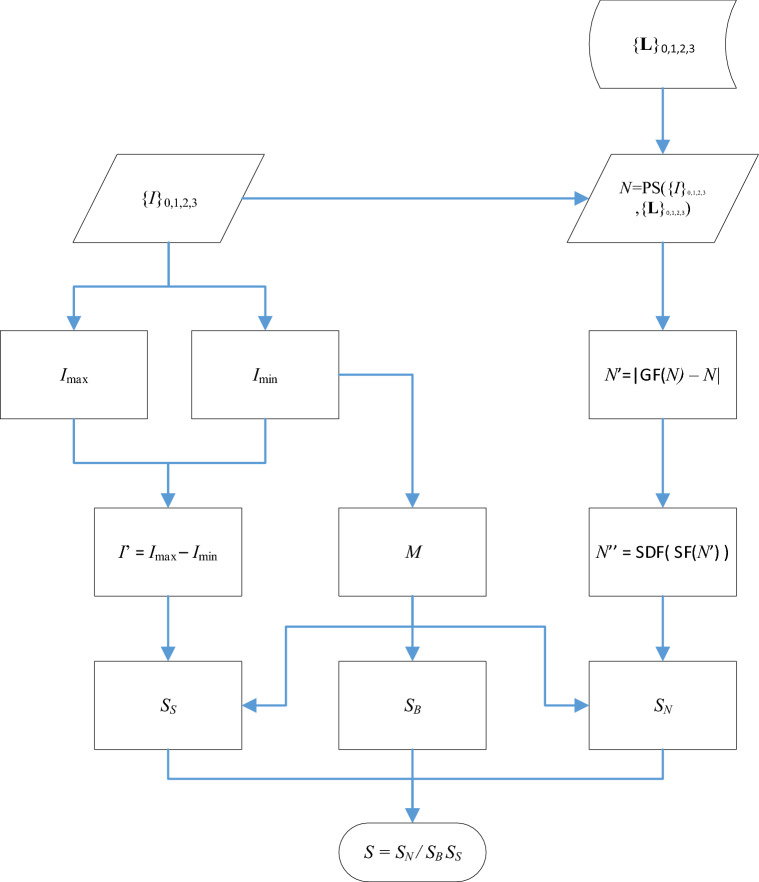
Flowchart to demonstrate the structure of the algorithm. For simplicity, this assumes *either* visible or NIR lights are used, but not both. The various symbols used are defined in the main text of the paper as they are first used.

The overall algorithmic structure is illustrated by Fig. 4. The top part of the diagram shows the set of four raw images, $\left\{ I \right\}_{0,1,2,3}$, and corresponding light source vectors, $\left\{ {\mathbf{L}} \right\}_{0,1,2,3}$, used for the photometric stereo function (“PS”). For example, the first light source has direction vector ${\mathbf{L}}_{0} = \left[ {L_{0x} ,L_{0y} ,L_{0z} } \right]^{T}$ from the centre of the sample and this generates an image $I_{0}$, which is an array of intensity values. Both the raw images and surface normals $N$ will be used for the computation of the metrics. For simplicity, only the $z$ (vertical) component of the surface normals are used.

##### Border

Consider first the process to compute the border metric. At the heart of this is the method to segment the part of the image containing the sample from the rest of the image. To do this, the well-established Otsu thresholding method^15^ is invoked. This automatically selects a threshold value whereby all pixels with an intensity above this are assumed to be “background” (since the ground appears lighter in the image than the actual faecal sample). Rather than applying the threshold to any single raw image, the algorithm is applied to image $I_{{\min}}$, which takes the minimum of the four raw image intensities for each pixel. This reduces bias towards a particular light source direction and was experimentally shown to be more robust than using, say, the mean intensity.

**Figure 5:**
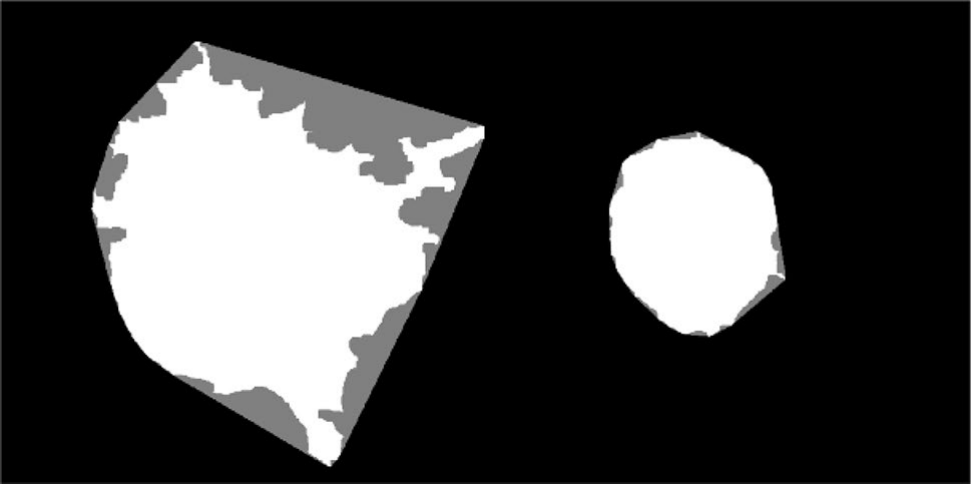
Segmented regions and convex hulls for the two extreme samples in Fig. 3. The solid white areas are masks, while the areas enclosed in grey are the convex hulls. The border scores for these samples are 0.74 and 0.14 respectively.

The validity of this clearly depends on the surface from which the sample is captured so may need revising for application in the field. Direct application of the Otsu method results in a few spurious regions such as where faeces has split up on the ground or where there are bright spots in the image. These are cleaned up by a few standard morphological operations: filling holes, eroding the perimeter and extracting the largest contiguous region of the image. The result of this is a “mask” image $M$ that shows the approximate region of the sample. This takes the form of a binary image of same dimensionality as the original (4 megapixels) where pixels have value “1” if they are part of the faecal sample and “0” otherwise. The white areas in Fig. 5 are examples of extracted masks. In future work, a *mask regions convolutional neural network*^16^ could be applied here to reduce the effect of foreground–background similarities further, thus making the method reliable in less controlled conditions.

The border metric itself is extracted using the “solidity” measure. First, the convex hull of the segmented image is found. This is the smallest possible fully-convex polygon capable of containing the entire sample. The solidity is then defined as the proportion of pixels in the convex hull that are also in the mask. This is illustrated in Fig. 5 where larger grey areas force a lower score: the sample with a smoother edge has a convex hull that more closely matches the mask. The border metric, $S_{B}$, is simply one minus the solidity, where in general, a higher score indicates a more irregular border. All scores (except the total score) are then normalised such that the maximum value is scaled to 1.

##### Shininess

**Figure 6:**
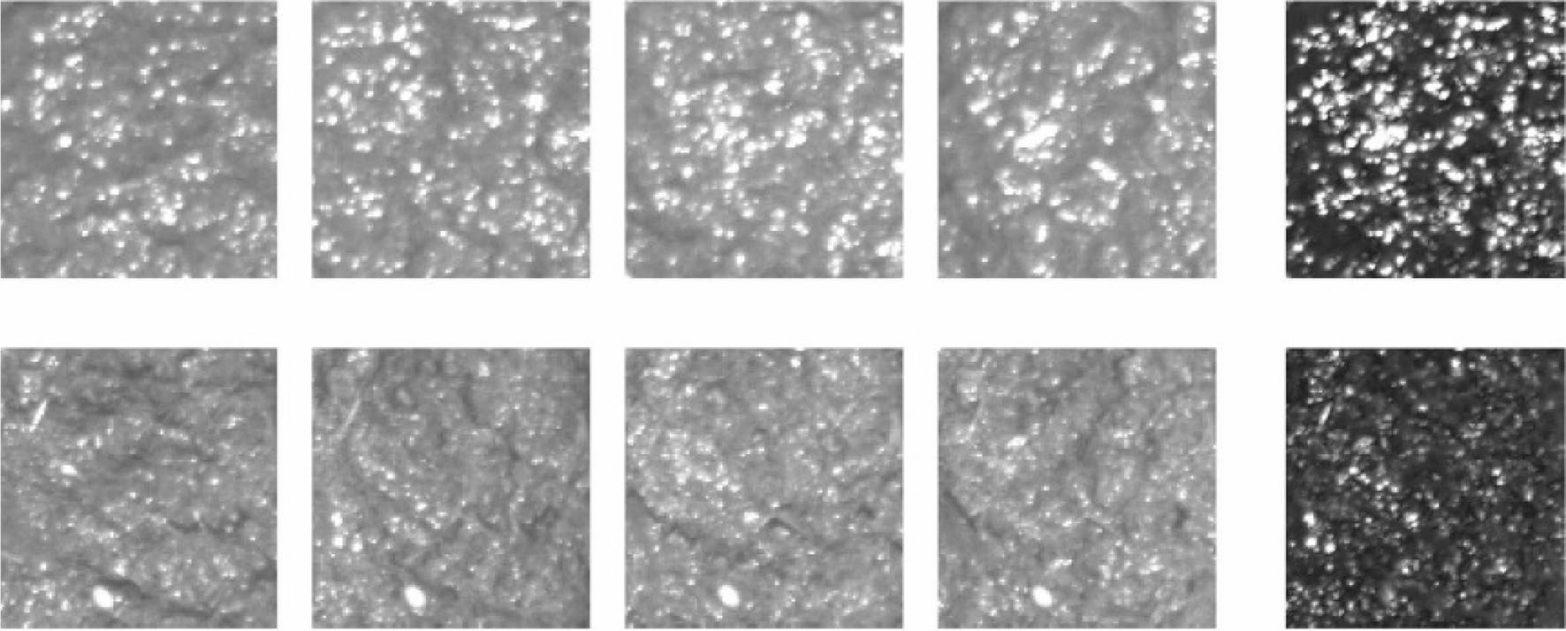
Portion of the raw images shown in Fig. 3 for to each of the four visible light sources with the difference between maximum and minimum intensity for each pixel shown to the right. The low and high scoring samples are on the top and bottom row respectively. The shininess scores for these cases are 0.84 and 0.53 respectively.

The left side of Fig. 4 shows the progression towards the shininess metric. The principle here is that shiny surfaces will exhibit a greater number of specular highlights: that is, there will be more regions of the image that appear to show strong direct reflections from the lights. This is demonstrated by Fig. 6: there are far more specular highlights in the low scoring sample.

The adopted method to quantify this is to consider that, for a given point on the sample, a specular highlight is unlikely to appear with more than one light source. Therefore, it can be expected that, of the four intensity values recorded for a given pixel, one will be much brighter than the other three in the presence of specularity. Let the highest of the four intensities be $I_{{\max}}$ and the lowest be $I_{{\min}}$. Consider now the difference, $I^{\prime} = I_{{\max}} - I_{{\min}}$. High values of $I^{\prime}$ indicate specularities and, therefore, shininess. An illustration of how $I^{\prime}$ is related to shininess is shown to the far-right of Fig. 6. The shininess metric, $S_{S}$, is then taken as the mean of the new image, $I^{\prime}$, for all pixels in mask $M$.

##### Normal

The right-hand portion of Fig. 4 describes the normal metric calculation. The key principle here is that more high-frequency features in the surface normal data corresponds to greater roughness. This motivates a two-step process. The first step aims to highlight such features by subtracting the normal data from a Gaussian-smoothed version of itself as shown in the top rectangle of the right-hand side of Fig. 4:

2 $$ N^{\prime} = \left| {{\text{GF}}\left( N \right) - N} \right| $$

where GF represents the Gaussian filter operation. The motivation for this approach is that the Gaussian filter has a blurring effect on the image: maintaining low frequency data and diminishing high-frequency. The difference between this and the original thus leaves only the high frequency data, which is the indicator of roughness. By simple trial-and-error, the Gaussian standard deviation of 5 pixels seemed to give qualitatively best results although this is not necessarily completely optimised. A different, but related, method to this was used previously for human skin melanoma detection^17^. An alternative would involve the Fourier transform but at greater computational expense.

**Figure 7:**
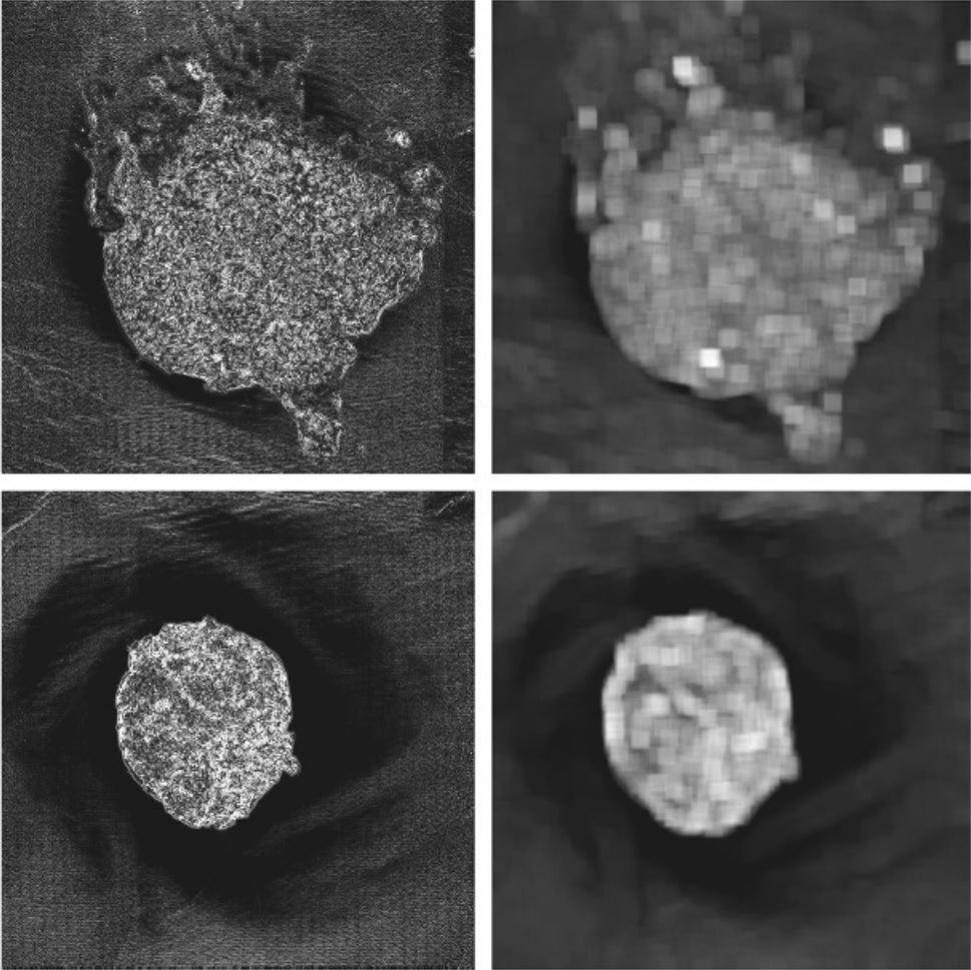
Left: images ($N{^{\prime}}$) after the first stage of normal metric computation using Eq. (2). Right: images ($N^{\prime\prime}$) after standard deviation filter applied. For this particular case, the normal metric scores are 0.73 and 1.

A median smooth filter, “SF”, is then applied in the next step where each pixel is replaced with the median of a 20-neighbourhood local median (higher weighting for centre pixels). As well as removing noise, this also smooths over spurious regions of small highlights in the image. Most importantly, the standard deviation filter, “SDF”, is then applied to obtain image $N^{\prime\prime}$. This acts in a similar way to the median filter except that the local standard deviation in $N^{\prime}$ is used to form a new image instead of the median. In this case however, there is no centre-weighting and a larger neighbourhood consisting of a circle of radius 51 pixels is used. The motivation for this is that firmer samples appear rougher in the image and should, thus, have greater local variations in intensity. A few other filters (e.g. entropy and range) and neighbourhood sizes were also tested with either similar or inferior results. Figure 7 shows the images resulting from this process. It is clear from Fig. 7 that high values are present for the rougher surface, as required. The final normals metric is then taken as the mean value of $N^{\prime\prime}$ for all pixels in mask $M$.

##### Total

The very final stage of the algorithm, shown at the bottom of Fig. 4, is simply to combine the metrics using Eq. (1). It is not immediately obvious that the three scores should be given equal weighting. In fact, it was empirically determined that weighting the shininess metric higher gave superior results:

3 $$ S = \frac{{S_{N} }}{{S_{B} S_{S}^{2} }} $$

This improvement may be due to an inherent feature of the shininess properties that shininess affords a generally more robust metric, or that values must be forced apart by raising the metric to a power. For the results in the next subsection, Eq. (3) was used to combine metrics, noting that this is not necessarily completely optimised.

### Machine learning for feature detection

This section describes how a machine learning algorithm was employed to classify images according to the presence, or otherwise, of large fibre particles or corn kernels in the samples. Classification was limited to these two types for simplicity. However additional types of feature could be added in the future using the same learning framework.

Deep learning^18^ is a ubiquitous method in modern computer vision due to its exceptionally high performance in solving previously intractable problems. Its main drawback is the need to “train” the system using a great many labelled samples. Deep convolutional neural network methods construct huge hierarchies of low, medium and high level image features based on a large number of labelled images. The distribution of these features for given image classes are then used to distinguish images or regions of certain objects. Unfortunately, it is often unfeasible to acquire the number of images needed per class for a given project/application.

Many recent contributions in the field overcome this challenge using the principle of *transfer learning*^18^. In this case (slightly simplified), the low and medium level features are used from pre-trained networks that contain a wide range of mostly unrelated images and the system is trained for the higher-level features using a smaller and more focused image database of relevant data.

**Figure 8:**
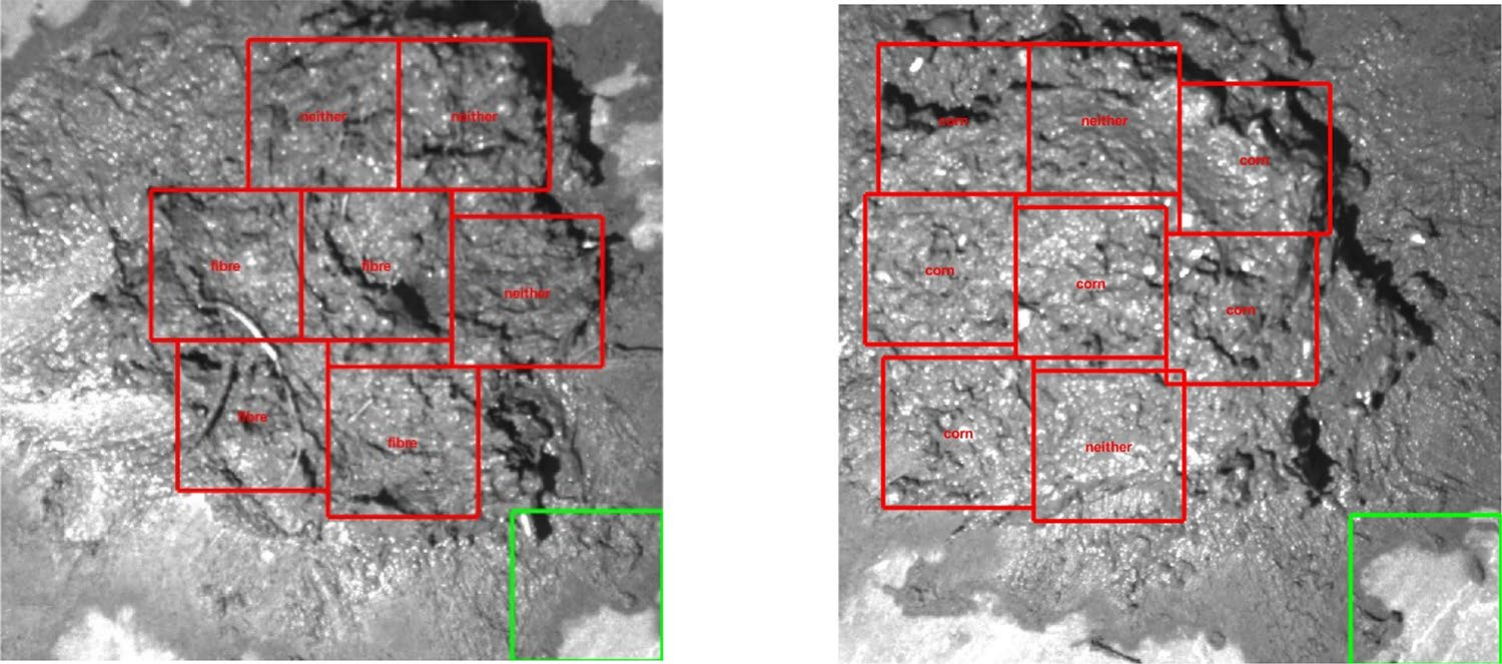
Example of manually labelled training data where each square is labelled either “neither”, “fibre”, “corn” or “both” (the lower-right box is a result of the MATLAB graphical user interface developed for annotation).

**Figure 9:**
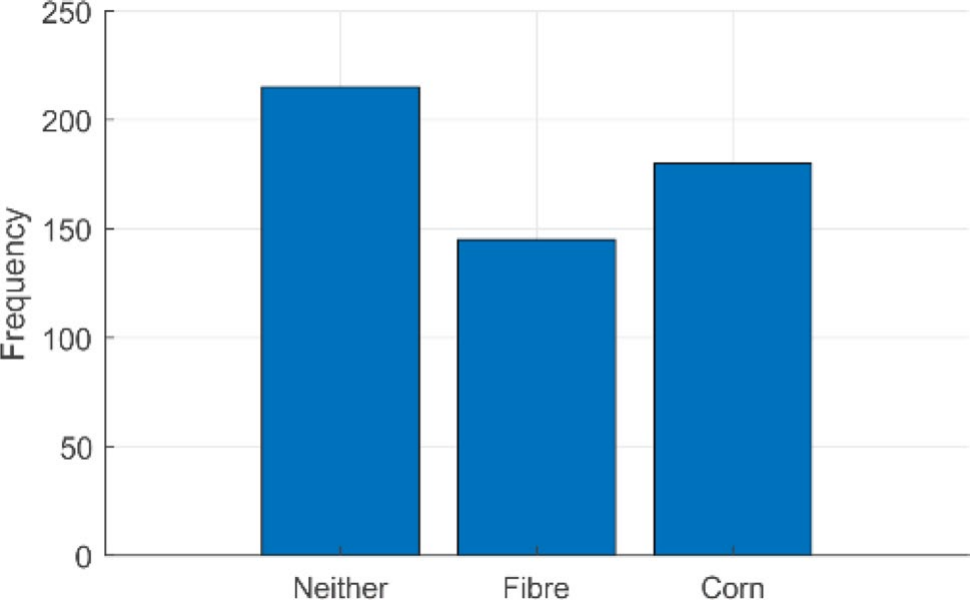
Distribution of training sub-images (neither: 215, fibre: 145, corn: 180).

For this study, transfer learning was used on 100 *soil* samples (to simulate real samples). The image database consisted of roughly equal numbers of samples without fibres or corn kernels, samples with fibres (extracted from celery) and samples with corn kernels. All soil samples had some water added to match a real faecal sample, but to varying degrees to simulate real variations in viscosity. The collected images were manually broken down into sub-images and labelled accordingly. Figure 8 shows a few representative examples. In practice, there was a slightly uneven distribution of class samples for training. Figure 9 summarises the numbers of sub-images used for each class.

Transfer learning was applied using MATLAB, which was also used to test the classification accuracy. A variety of network architectures were applied with a small, but significant variation in classification rate. The overall-best performing network was Resnet-101^19^ so all results presented in this paper use that architecture. Experiments were carried out using the standard MATLAB implementation of Resnet-101 with only the fully-connected layer and the classification layer changed, as necessary to reflect that only three possible output classes should be allowed (“corn”, “fibre”, “neither”). It is likely this gave best results since the network was designed to minimise over-fitting in relatively small datasets. However, it should be noted that developing a new or optimised network was not the focus of this paper and experiments were restricted to use those architectures already implemented in the MATLAB Deep Learning Toolbox such as AlexNet, GoogLeNet and ResNet-50/101^20^. The classification algorithm expects just a single image input (recall there are eight images from the PS rig—four from visible light and four from near-infrared light) so it was necessary to construct a final image from one or more of the raw images. The results of different image combinations are shown in the next section.

## Results and discussion

### Scoring using the three metrics

**Figure 10:**
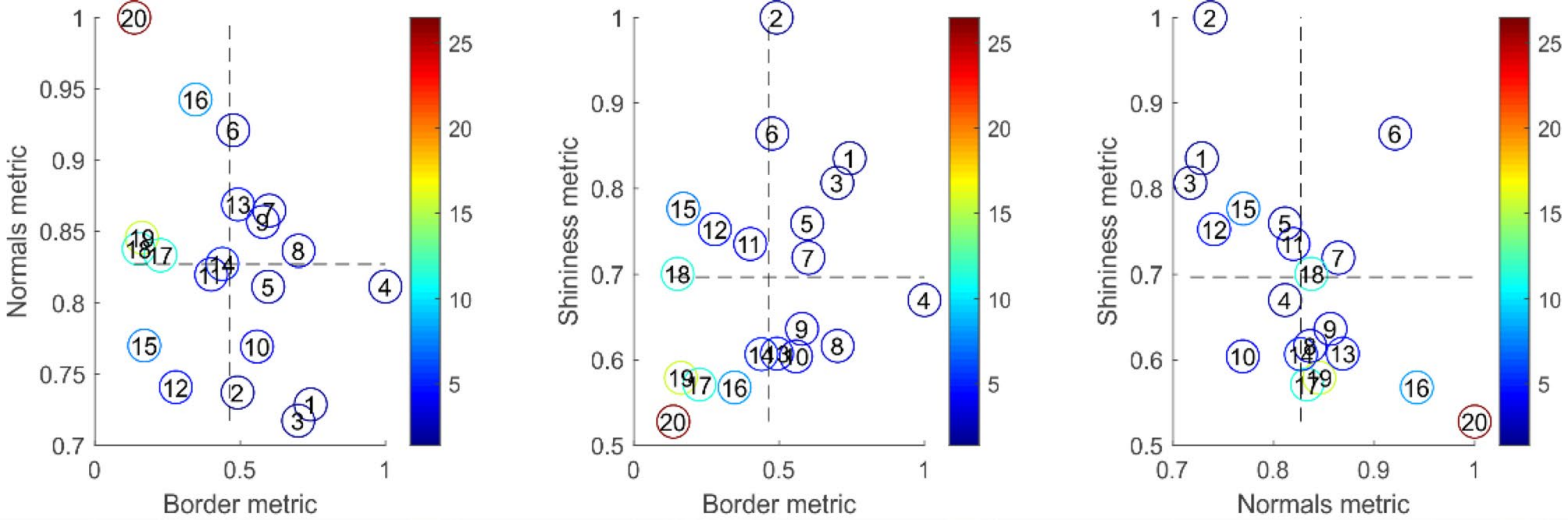
Distribution of metrics for all 20 samples. The colours indicate the total score while the numbers show the rank of the sample's total score compared to others. The data point ranked 1 (i.e. lowest of all scores) indicates the left-hand sample from Fig. 3, while the point ranked 20 is the right-hand sample of Fig. 3.

Table 1 summarises all metrics for the two extreme cases shown in Fig. 3. This shows good spread for each metric. Figure 10 shows the distribution of the metrics for all 20 samples collected at CEDAR. This shows that samples are well-spaced in score-space. As expected, given the arguments leading to Eq. (1), results show negative correlation between the normal score and both shininess and border, but positive correlation between shininess and border scores.

**Table 1:** Summary of individual metrics for the extreme cases.

Sample	Border	Shininess	Normal	Total
Left in Fig. 3	0.74	0.84	0.73	1.4
Right in Fig. 3	0.14	0.53	1	26

**Figure 11:**
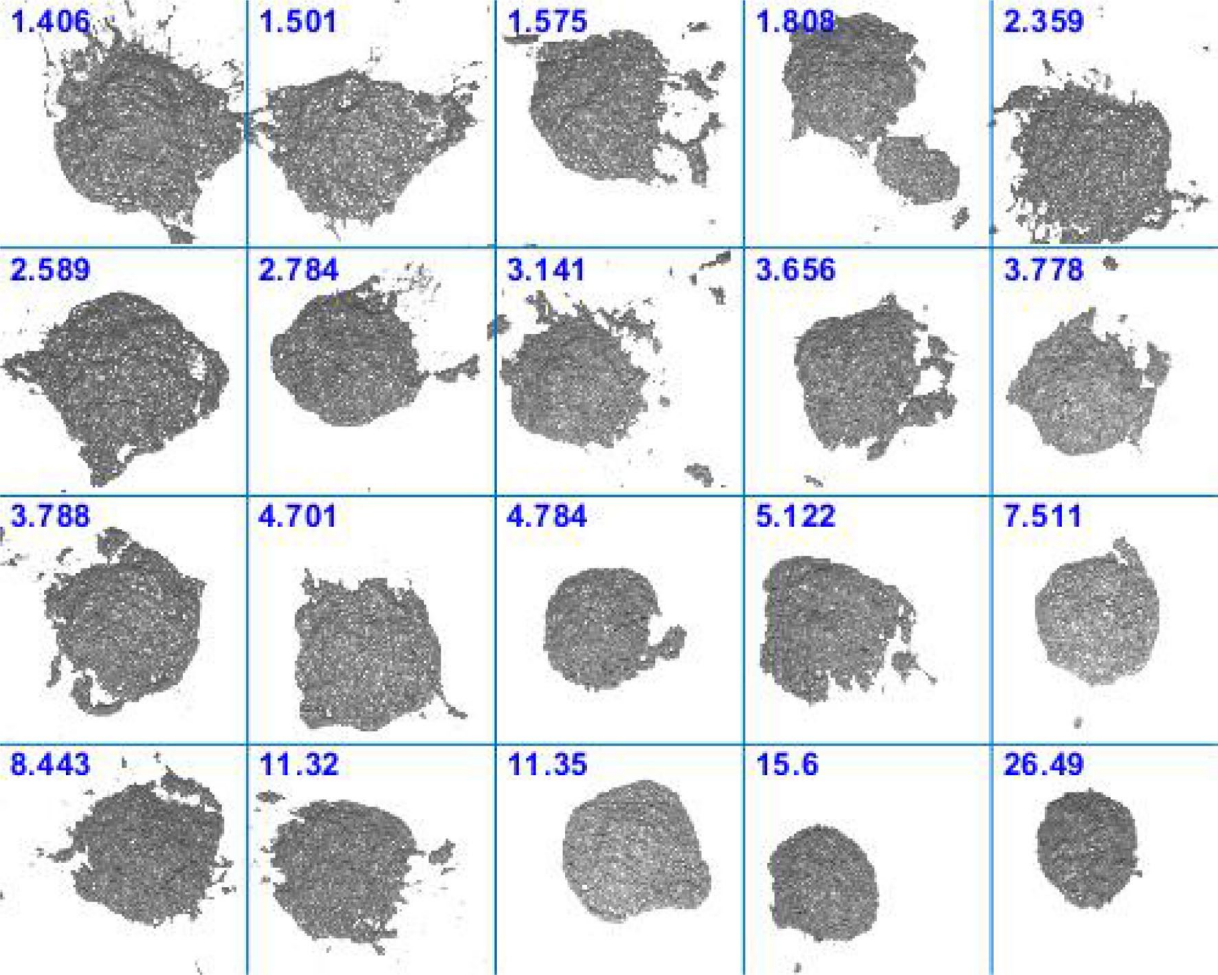
Images of samples according to calculated total score (score values above-left of each sample).

The full set of images is shown in Fig. 11 in order of total score. The method appears to have been successful in placing the samples in an order that closely matches the manual scoring method. Given the relatively small variation among the samples, it is difficult even for a human to place these in an exact order. There are certainly no obvious mistakes in the ordering of the samples except perhaps that the fourth-lowest-ranked sample should probably have a higher score. This is a result of the sample splitting in two when dropped (it has by far the highest border metric) and would likely be addressed using a more repeatable drop process (see Conclusion).

### Corn kernel and fibre detection using deep learning

**Figure 12:**
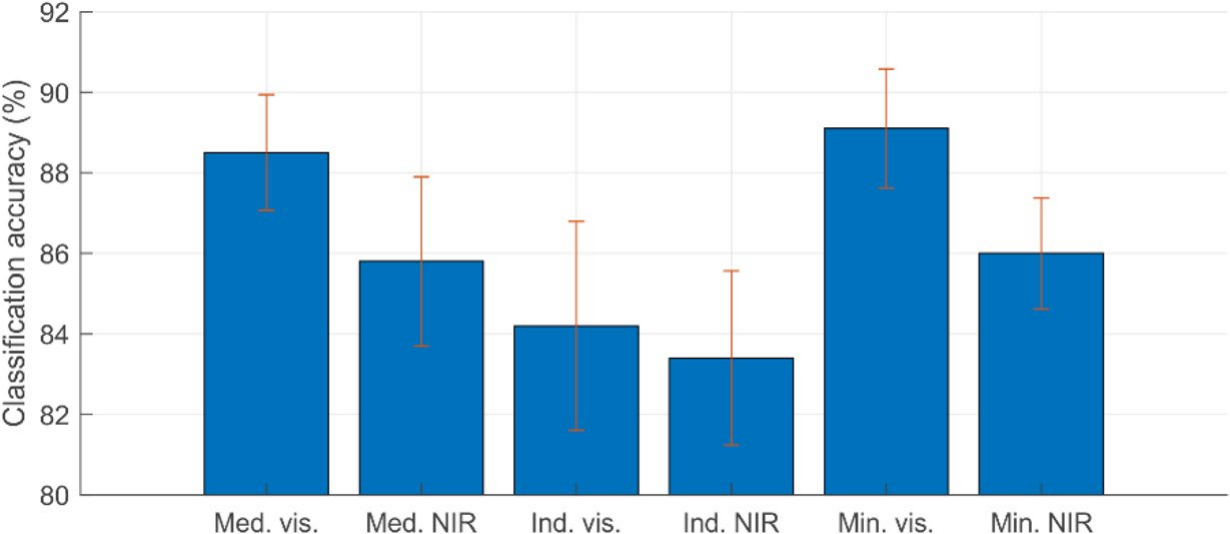
Classification rates using MATLAB's default training parameters for various image combinations. The whiskers indicate the standard deviations of accuracies when the test dataset images are changed.

Classification rates varied depending on how the raw images from the capture rig were pre-processed to form inputs to the neural network. The rates are summarised in Fig. 12. The most obvious way to combine the images was to simply take the mean intensity across all raw images (either all four visible images, all four NIR, or all eight combined). However, this gave very poor results, barely improving on statistical chance. It is thought that this poor result is due to the mean intensity having the effect of smoothing over useful information. More successful combinations are shown in Fig. 12.

Interestingly, excellent results were achieved using the median (“med. vis” and “med. NIR”) instead of the mean: presumably as this does not have the smoothing effect mentioned above and/or a reduced impact of outliers. It does however, seem better when just using the visible lights rather than NIR (though note the large standard deviations in test results). Results using individual images (“ind. vis.” and “ind. NIR” in Fig. 12) were unsurprisingly inferior while using the minimum intensity for a given pixel location gave best results of all. Results for the maximum intensities are not shown as this confounded the algorithm by emphasising specularities.

The fact that the results in Fig. 12 are so varied, and that the other network architectures considered only affected results by a few percentage points, reinforce the more general research^21^ that image quality and pre-processing are the key steps to optimisation. Fine-tuning the network optimisation parameters from the MATLAB default gave similarly small improvements although optimising the *mini-batch size* did yield results over 90% in certain cases when set to 30 or higher.

The highest recognition rates here are clearly very promising; especially considering that the manual labelling process was limited in that it did not permit much freedom in how the image was broken down (a mask regions convolutional neural network is more flexible^16^ but is reserved for future work). On the other hand, the samples used here contain more visually obvious features compared to real samples which would typically have finer and shorter fibres for example. Therefore, it is unlikely that the method in its current form would extrapolate to real-world data without any reduction in classification rate. This will be explored in further work.

## Conclusion

This research has proven the potential for the use of computer vision in the assessment of cattle faeces consistency and large particle content. The scoring method closely matched subjective analysis while the deep learning approach gave promising results in the detection of undigested corn and fibre. While the current data capture hardware is clearly not as convenient as, say a mobile telephone camera, it is nevertheless portable and cheap enough for realistic usage on a farm and diminishes the need for regular samples to be transferred for laboratory analysis. While the current device shown in Fig. 1 is clearly only at prototype stage, this could easily be made more compact and transportable. In particular: the computer could be replaced with a low-cost embedded system (using a Raspberry Pi^22^, for example); the desktop power supply can be replaced with a simple fixed-voltage unit; and the legs could be made foldable. These steps would allow the entire system to fit into a padded briefcase-style container.

**Figure 13:**
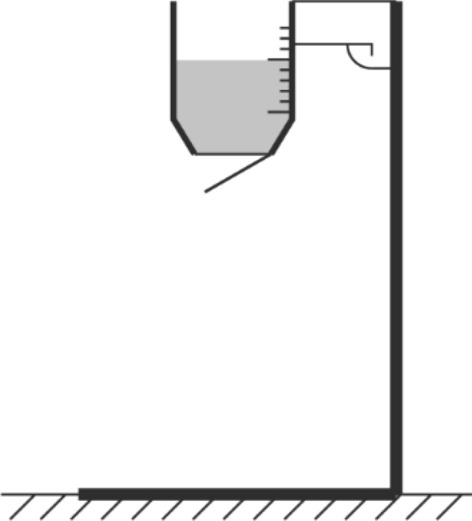
Schematic design of a device that would make the dropping process more convenient and repeatable. The device has a fixed height, has a detachable cup with scale to scoop the correct amount of sample and a trigger to release an opening below.

Despite the clear outcomes of the research, there remain a few questions/tasks necessary to consider during any follow-on research to make the method more applicable:

- A greater range of fibre and corn sample training images would improve robustness.
- The scoring algorithm should be tested on a greater range of samples (i.e. those scoring 1 or 5 in the literature's scoring method^13^) and mapped to more established metrics.
- The scoring system would be more reliable if the process for dropping the samples were made more convenient and repeatable: e.g. by releasing the sample from some device, such as that suggested in Fig. 13.
- It may prove that a *deep regression network*^21^ is able to enhance the scoring by better learning the means by which human experts grade the samples.
- As mentioned in the introduction, it is hoped that the method can be calibrated against better-established technologies, such as visual assessment or wet screening of faeces to establish particle size distribution^6^. This would theoretically allow to attain the precision of such well-known methods using the more objective technology discussed in this paper.
- Other features such as “bubbles”, which are indicators of abnormal hindgut fermentation of starch, could be investigated to incorporate into a feature detector.
